# A Signal Processing Method to Explore Similarity in Protein Flexibility

**DOI:** 10.1155/2010/454671

**Published:** 2010-12-20

**Authors:** Simina Vasilache, Nazanin Mirshahi, Soo-Yeon Ji, James Mottonen, Donald J. Jacobs, Kayvan Najarian

**Affiliations:** ^1^Department of Computer Science, Virginia Commonwealth University, Richmond, VA 23284, USA; ^2^Department of Computer Science, Bowie State University, Bowie, MD 20715, USA; ^3^Department of Physics and Optical Science, University of North Carolina at Charlotte, Charlotte, NC 28223, USA

## Abstract

Understanding mechanisms of protein flexibility is of great importance to structural biology. The ability to detect similarities between proteins and their patterns is vital in discovering new information about unknown protein functions. A Distance Constraint Model (DCM) provides a means to generate a variety of flexibility measures based on a given protein structure. Although information about mechanical properties of flexibility is critical for understanding protein function for a given protein, the question of whether certain characteristics are shared across homologous proteins is difficult to assess. For a proper assessment, a quantified measure of similarity is necessary. This paper begins to explore image processing techniques to quantify similarities in signals and images that characterize protein flexibility. The dataset considered here consists of three different families of proteins, with three proteins in each family. The similarities and differences found within flexibility measures across homologous proteins do not align with sequence-based evolutionary methods.

## 1. Introduction

Proteins are complex biomolecules, combining the structure of a long folded polypeptide chain with the underlying dynamics that contribute to function. The relative rigidity and flexibility along protein chains may provide insight into evolutionary and regulatory mechanisms controlling function. Besides traditional experimental measures that can be attributed to flexibility, such as crystallographic B-factors or NMR S^2^ order parameters, computer models exist that attempt to quantify protein flexibility [1]. One unique methodology, termed the Distance Constraint Model (DCM) [2], considers various interactions, such as covalent bonds, hydrogen bonds, and local residue conformational states, and models these as a network of distance constraints. A large number of Quantitative Stability and Flexibility Relationships (QSFR) are output of a minimal DCM (mDCM), where rigidity/flexibility properties of an underlying distance constraint network are obtained through graph rigidity algorithms that allow mechanical properties to be calculated. 

Proteins with high-sequence identity and high structural similarity share a common evolutionary relationship and have similar structure and function [3]. However, orthologous proteins may also have subtle yet important differences due to changes in regulation, cellular environment, among other factors. The DCM produces copious output measures for each protein studied, which are typically analyzed by graphing and then visualizing the data. Comparative studies that quantify the similarity of proteins based on flexibility measures using standard clustering methods have demonstrated deviations from those obtained from sequence alignments [4]. Despite differences that were found in clustering methods, visual inspection shows many features are qualitatively similar, while there are subtle differences in localized regions. Since subtle differences can have a dramatic effect on how a protein functions, it is necessary to analyze the data across global and local scales in quantitative detail in order to accurately detect regions of protein similarities and differences. In this regard, image recognition offers a possible option toward such an analysis. For instance, wavelets are capable of decomposing, displaying, and analyzing signal patterns in space and scale domains. Wavelets allow exploring the relation between frequency and space characteristics of a signal and are widely used to investigate the near and far similarities in terms of location in sequence [5].

In recent years, wavelet analysis and image processing algorithms have been increasingly applied to bioinformatics. Doolittle [6] described wavelet transform as an exceptional tool for feature extraction and multiresolution signal analysis. Image processing methods have been used for content-based image retrieval, and image registration methods to measure the dissimilarity of proteins [3]. In addition, discrete wavelet transform has been employed to discover functional similarity of proteins with low identity based on various substitution models [7]. Given the successes that wavelets have provided up to now, it is worthwhile to apply wavelet analysis (for the first time) on the flexibility measures generated by mDCM. 

For this study, three proteins from thioredoxin (TRX) calmodulin (CaM) and CheY (CHY) protein families were selected as a representative dataset. On this data, wavelet analysis is employed to explore the flexibility characteristics of three proteins from each of the three families. In prior work [8], the sequences were aligned using standard structural alignment methods. However, an applicable question is whether one can use the flexibility characteristics as a determinant for the alignment. It is possible that aligning proteins using intrinsic flexibility properties will provide insight into functional aspects tied to protein dynamics, rather than structure. Therefore, an image registration method is employed in this work to compute the similarities. A problem that occurs when comparing sequences of different lengths is the formation of gaps. The gaps are represented as large values that are off scale.

The rest of the paper is organized as follows: the proposed methodology is described in Section 2, starting with the description of the dataset of study, proposed method, and then, the steps of image registration. The results of the proposed method are presented and discussed in Section 3.  Section 4 presents the conclusion and future work.

## 2. Methodology

### 2.1. Dataset

For the purpose of this study, the particular selection of proteins is not important with respect to biological function. Nevertheless, all proteins selected have much relevance in biological function in humans and/or other species. Here, nine proteins are considered, where three proteins have been selected in three different families, associated with thioredoxin (TRX), calmodulin (CaM) and CheY (CHY).

The proteins in the thioredoxin family are essential for vital functions in different organisms from mammals to bacteria [9]. The TRX proteins are redox regulators to maintain intracellular target proteins in a reduced state. They are relatively small (~110 amino acids) and are well characterized structurally and have been studied to considerable extent using the mDCM [4, 8]. The mDCM parameters are generally determined by fitting calorimetry data. In this case, the parameters were determined form *E. coli* protein (PDB code 2TRX), and the same parameters were used to generate QSFR for orthologous thioredoxins from *Anabaena* species (1THX), *Chlamydomonas reinhardtii* (1EP7), and *Spinacia oleracea* (1FB6). Further details of the mDCM methodology and protein structure processing can be found in [4, 8]. 

The proteins in the calmodulin family are essential for regulation by intracellular signaling [10]. The family plays a key component for modulating muscle physiology through calcium binding, and it interacts with over 300 different targets [11] that play a role in inflammation, apoptosis, immune response, metabolism, and memory. Calmodulin is a moderately small protein (~150 amino acids) with two domains connected by a linker region. The mDCM parameters have been obtained by the usual method of fitting to heat capacity data (to be published elsewhere). 

The proteins in the CheY family have ~130 amino acids. CheY is part of the bacterial chemotaxis signal transduction pathway [12]. CheY is phosphorylated (activated) by CheA, which results in a 20-fold increase in affinity for the flagellar switch protein FliM [13]. Upon CheY/FliM association, the flagellum switches to a clockwise rotation which results in random tumbles that reorient the bacterium. The mDCM parameters were previously obtained by fitting to heat capacity curves as usual, and by making a judicious selection of parameters for some proteins without heat capacity data, as described in [14]. 

The mDCM is an ensemble-based method, meaning that in this method several distance constraint networks are analyzed. The rigidity and flexibility properties change depending on how distance constraints are distributed within the network. Such network fluctuations occur because various interactions such as hydrogen bonds form and break. As such, it happens that certain sets of residues become rigidly or flexibly correlated. The differences that are found between realizations are in accordance to thermodynamic equilibrium probability. Since a given residue may fall in a region that is rigidly or flexibly correlated with certain probabilities, the mDCM provides average values of quantities, and for these quantities, the fluctuations occur over the ensemble of realizations.

**Figure 1:**
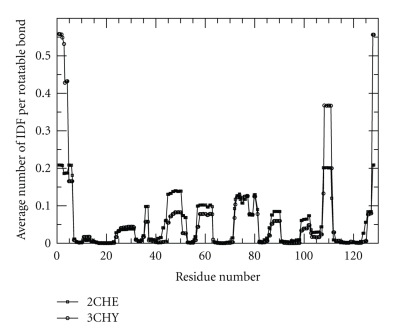
Example of two IDF signals without gaps.

For each protein, wavelet analysis is applied to two different flexibility metrics. The first flexibility metric considered in this work quantifies the average number of independent degrees of freedom found within the protein, and their location along the backbone. This is a one-dimensional signal that has the sharpest features of all flexibility metrics that the mDCM calculates. An instance of such a metric is given in Figure 1, comparing 2CHE with 3CHY. Observations show that many regions are essentially identical, and other regions have noticeable qualitative similarity, yet distinct features. The idea for applying signal analysis will be to account for these subtle details in differences and similarities. 

The second metric, called the rigidity susceptibility, is a symmetric two-dimensional image represented by a square matrix that quantifies the propagation of rigidity and flexibility fluctuations through the protein. When a contiguous region is found to be rigid, all residues are mutually rigid within this region and are defined as rigidly correlated to one another. On the other hand, if a single flexible region is determined to span across a group of residues that are flexible, they are defined as being flexibly correlated. Note that flexible regions contain rigid components. For example, consider a chain necklace where each link is rigid, yet there is flexibility between each link. A single flexible region indicates that generic motion in one part of the region will propagate throughout the identified region. The flexibly and rigidly correlated residues are uniquely defined as described thoroughly in a focused review [15]. 

**Figure 2:**
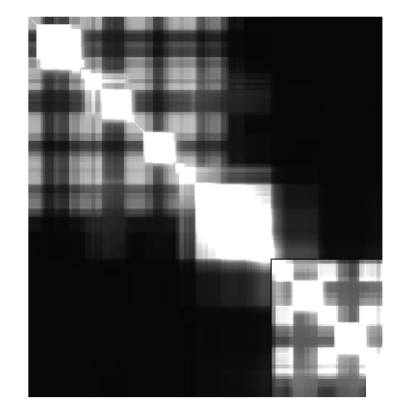
Susceptibility (SUS) signal image for protein 1RFJ belonging to family CaM.

A random variable, *n*_*jk*_ is introduced to indicate the rigidity state between residues “*j*” and “*k*”. If *n*_*jk*_ = −1, both residues are part of the same rigid region. If *n*_*jk*_ = 1, both residues are part of a flexibly correlated region. For instance, if residue “*j*” and residue “*k*” are mutually rigid, the symmetric matrix elements at (*j*, *k*) and (*k*, *j*) are *n*_*jk*_ = *n*_*kj*_ = −1. On the other hand, if both residues are flexibly correlated, the matrix elements are assigned *n*_*jk*_ = *n*_*kj*_ = 1. It is possible for a residue neither to be mutually rigid with other residues nor to be flexibly correlated with other residues (i.e., a residue can be flexible, but not correlated). This situation always occurs within dangling ends, which are not part of a loop. In that case, the assigned matrix elements are set as *n*_*jk*_ = *n*_*kj*_ = 0. The standard formula for variance of a random variable: *𝒳*_*jk*_ = *𝒳*_*kj*_ = 〈(*n*_*kj*_)^2^〉 − 〈*n*_*kj*_〉^2^ is applied to get the susceptibility in rigidity along the backbone where 0 ≤ *𝒳*_*jk*_ ≤ 1, and the matrix elements maintain all information about correlations in how the rigidity and flexibility propagate through the protein. An illustration of a rigidity susceptibility matrix is given in Figure 2. 

For both types of flexibility metrics described above, the idea of characterizing the state of a residue or comparing a pair of residues was implied. However, in the actual calculations, the PSI and PHI angles are the elementary units that are being characterized. These torsion angles along the backbone are within each residue, flanking both sides of the alpha-carbon atom. Hence, each residue has two values. Meaning, a protein of 100 residues (i.e., amino acids), will have 200 entries, associated with the PHI and PSI angles per residue. Details are explained in prior works [1, 8, 15]. Many different types of flexibility metrics output from the mDCM. The main reason for selecting the metrics

- density of Independent Degrees of Freedom (IDF)
- rigidity susceptibility (SUS)

is because they are both positive definite quantities which make the signal processing easier, and, because of the juxtaposition that these two quantities offer. Essentially the IDF signal is a highly varying function, while the rigidity susceptibility provides the weakest spatial variation among the image-like quantities that are calculated. Furthermore, the first measure is a signal, and the second measure is an image.

### 2.2. Proposed Method

**Figure 3:**
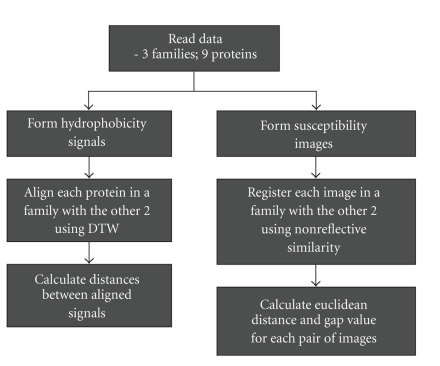
Outline of the proposed method.

The schematic diagram shown in Figure 3 outlines the methodology employed in this paper to analyze the flexibility characteristics of nine proteins three from each of the three protein families a total of: TRX, CaM, and CHY. The generality of the method is not restricted by this particular choice of flexibility metrics or proteins. It is important to notice that for symmetric images, only the upper half of the image needs to be processed, although the entire image was processed in generating the results herein. 

 The proposed method incorporates Dynamic Time Warping- (DTW-) derived alignment techniques; the Fourier transform and 2D signal analysis in the form of image registration. Image registration is performed with the aim of achieving maximum similarity between a pair of 2D signals. Initial images are not equal in size; therefore, resizing of the image is performed prior to registration. Registration is performed using our modified and customized version of similarity registration method [16], implemented in MATLAB. This algorithm is described in detail in Section 2.3. Using this transformation ensures appropriate resizing of the image without distorting the image elements. In particular, straight lines are maintained and parallel lines remain parallel.

### 2.3. Image Registration

Image registration is a method that allows mapping of images through proper transformations. Registered images then can be analyzed and compared using computational methods. Image registration methods have the capability to transform a set of information to a corresponding set of information in identical coordinate systems. In practice, image registration is essential for comparison and integration of the data that are obtained from different measurement methods. Image registration methods are divided into two main categories: manual image registration methods and automated image registration methods. Applications of automated image registration methods are in areas such as medical scanning, astronomy and mutual information shared by images. 

As for manual image registration methods, manual landmark is the technique that is extensively used in commercial proteome. The accuracy of registration is improved by automated feature selection; however, at the same time it results in higher complexity of computations and time. To simplify the computation and to reduce the time complexity of the method, manual landmark was used in this study. Figure 2 depicts the susceptibility values for pairs of sequences. Since the studied proteins have different amino acid sequence lengths as well as varying high/low values of susceptibility, image registration was applied for analysis and for discovery of the similarities. 

In this section, the main steps of the image registration algorithm are described. First, in order to estimate the values for the regions of discontinuity, the algorithm uses interpolation by averaging the neighbors. The interpolation translates into identifying the points with missing values and assigning them the arithmetic mean value of their nonmissing neighbors. In the resizing of images, bicubic interpolation is involved. This type of interpolation exhibits smoother transitions and the resampled images have few interpolation artifacts. 

Following that, images are transformed using similarity transformation. In such a method, the unregistered image is registered and resized to the dimensions of the base image without introducing distortion in the registered image elements–straight lines are maintained and parallelism is kept. Then, the similarity measures are calculated as the average of the distances between a point in the base image and its corresponding point in the registered image. This measurement is used for the positions in the image where both the base image and the registered image have defined values

(1) $$d\left(im_{i},im_{j}\right)=\overset{x=m,y=m}{\underset{x=1,y=1}{\sum }}\frac{{\left(im_{i}\left(x,y\right)-im_{j}\left(x,y\right)\right)}^{2}}{m^{2}},$$

where *im*_*i*_ and *im*_*j*_  are signal images of dimension *m* × *m*, *x* and *y* are pixel coordinates in the image and *im*_*i*_(*x*, *y*), *im*_*j*_(*x*, *y*)  are the grey level values of the pixel at coordinates (*x*, *y*) in image *im*_*i*_ or *im*_*j*_, respectively. In addition, a measure for the influence of gaps in alignment is introduced and this measurement is the number of gap values normalized by the size of the susceptibility image.

To summarize, in order to determine protein similarity within the three proteins in each of the three families using image analysis, the methodology is as follows: each of the three susceptibility images is used as base and the other two images are registered to it by selecting control points and using automatic registration via the maximum similarity method. Subsequently, two measures are calculated between the original image, which served as base, and the other two. As such, we will have six registered images within each family and six sets of measures (between one of the three base images and one of the two images that were registered to it). The two measures are: Euclidean distance calculated between the pixels in the image which have real values and a normalized count of gap values for the image pixels in which either the original image or the registered image contains a gap value.

After calculating these two measures they are averaged for one pair of images as follows: if *X* and *Y* are two susceptibility images, first *X* serves as a base and *Y* is registered to it, then the two measures of similarity are calculated. Following that, the roles are inversed and another set of two similarity measures is calculated. Finally, to obtain the measure of similarity between images *X* and *Y* arithmetic mean for both the Euclidean distances and normalized count of gap values are computed.

### 2.4. 1D Signal Analysis

For the analysis of signals, Dynamic Time Warping (DTW) method was used. DTW is a method that is capable of measuring the similarity between signals/sequences with different lengths as well as aligning them. Finding an optimal match between two given sequence, the method has numerous applications in processing of audio, video, and graphics. Speech recognition is considered the most common area of study related to DTW. DTW method discovers a path that needs to be continuous and expand over the entire length of the sequence. The recursive version for calculating DTW follows

(2) $$\begin{array}{cc} DTW\left(i,j\right) & =d\left(i,j\right)\\ & \;+\underset{}{\min\;}\;(DTW\left(i,j-1\right),\\ & \;\;\;\;DTW\left(i-1,j-1\right),DTW\left(i-1,j\right)), \end{array}$$

where *i* = 1 ⋯ *n*, *j* = 1 ⋯ *m* and *m*, *n* are the dimensions of the signals being measured, *d*(*i*, *j*) is the distance between two points in the signals *DTW* is calculated for

(3) $$d\left(i,j\right)={\left({\text{signal}}_{1}\left(i\right)-{\text{signal}}_{\text{2}}\left(j\right)\right)}^{2}.$$

Due to its recursive nature, the time complexity of the method, when using the above mentioned formula, exponentially increases with the length of the sequences that are being analyzed. Since the lengths of the output measures (2 per residue) of this study are larger than 200, a forward version of the formula is implemented in this research. The signals analyzed for each protein are the hydrophobicity signals and the IDF signals. The hydrophobicity signals are created by assigning a hydrophobicity value to each amino acid in the order given by the protein sequence. This hydrophobicity sequence/signal is then processed to extract the patterns that might exhibit similarities and differences among the proteins within a family. The hydrophobicity values for each amino acid were taken from [17].

## 3. Results

The results of applying dynamic time warping method to IDF sequences and hydrophobicity signals are shown in the table below and are organized for the three different families.  Table 1 presents the distances calculated for one-dimensional signals formed using IDF and hydrophobicity values.

**Figure 4:**
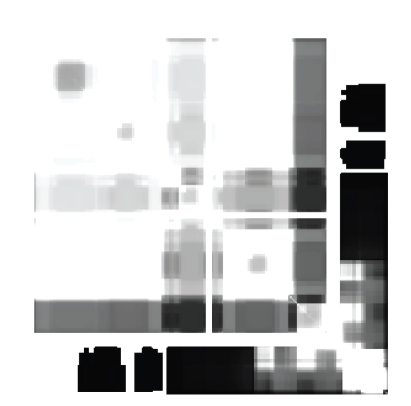
Registered image of 1TMY to 3CHY.

**Figure 5:**
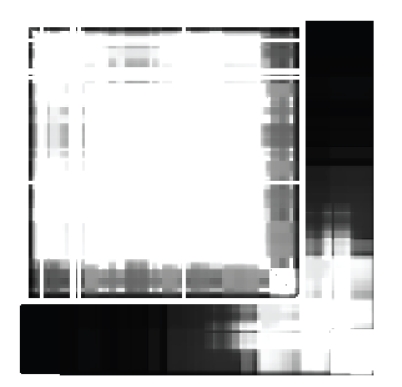
Registered image of 1FB6 to 1THX.

Figures 4 and 5 illustrate samples of the registered images between different proteins within the same family. Registration rescales and interpolates images with different sizes making their sizes equal for direct comparison. Registration puts the original image and the image to be registered on top of each other, using appropriate control points and provides a means to calculate a direct quantitative difference between the two images. The black and white pixels/areas in the registered images are interpreted in the same way as in the original images, that is dark regions correspond to regions lacking the property (e.g., susceptibility) and bright regions display regions that have high degrees of that property. 

When pairs of residues fall within a dark region, they are not dynamic. Meaning, they remain mutually flexible or rigid, with little fluctuations. Conversely, white areas are regions that are unstable mechanically, transitioning from flexible to rigid, and vice versa. Regions in flux are expected to be important to function because perturbations, such as ligand binding, can drive these “confused” regions to settle on being either rigid or flexible. In other words, these regions are mechanically more susceptible to allostery.

 As mentioned above, the registered images will allow calculating distances between two proteins. Tables 2, 3, and 4 present these image-based distances, both the Euclidean that presents mismatch information, and the gap measure, for all proteins within the three family. 

**Figure 6:**
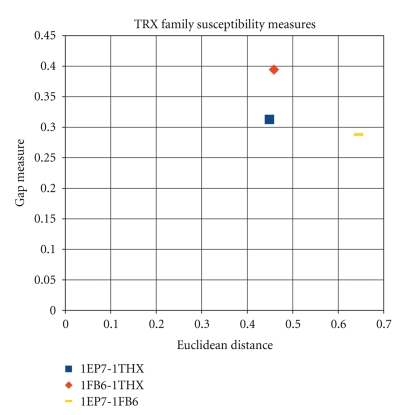
Plot of susceptibility measures for the TRX family.

**Figure 7:**
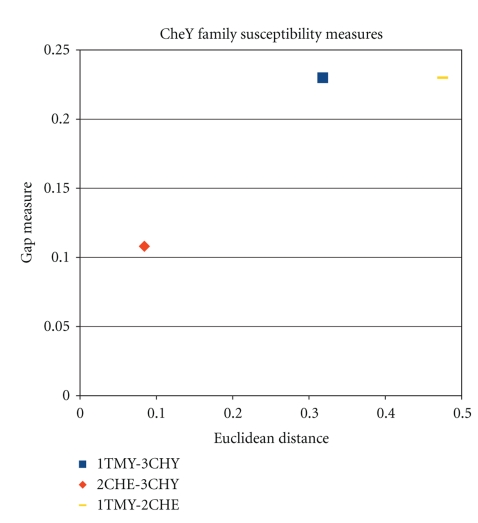
Plot of susceptibility measures for the CheY family.

**Figure 8:**
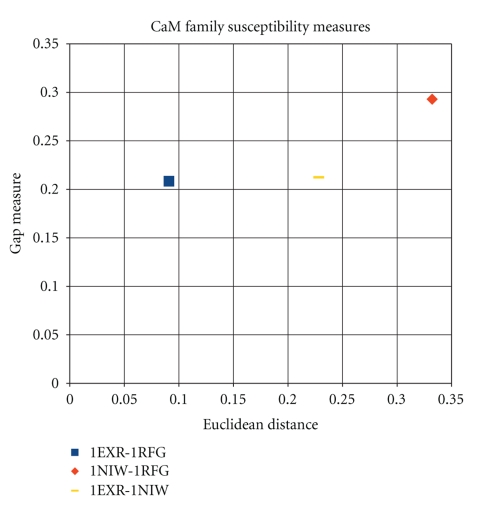
Plot of susceptibility measures for the CaM family.

Figures 6, 7, and 8, illustrate the results of the last three tables 2D scatter plots of Euclidean distances and gap measures between registered susceptibility images. In other words, for each family of proteins, the figures represent scatter plots of the two distance values calculated for each protein pair within a family.

## 4. Conclusion and Future Work

Signal processing methods were applied in order to quantify similarities and subtle differences that arise in flexibility measures between different members of a protein family. dynamic time warping, image registration, and the Fourier transform interpolation were utilized in order to match different protein signals to one another. The detailed Fourier decompositions and the registered images appear to provide a wealth of quantitative information; however, whether these results actually highlight distinctive attributes related to protein function requires further research.

 A much simpler question is to see whether the wavelet analysis allows for distinguishing between different pairwise protein comparisons. The joint measure that accounts for both gap and Euclidean distances between protein pairs is found to provide a sensitive measure. In previous works, there has been difficulty in quantifying similarity content contained in comparisons of pairwise protein flexibility characteristics. The encouraging aspect of the results presented here is that in all three comparative studies (one per family), it is found that the images that “visually look similar but have some distinct features” have been successfully decomposed quantitatively. Consequently, the signal processing method presented above is found to well-separate homologous proteins quantitatively (as shown in Figures 6, 7, and 8).

Further research on this approach, we propose to design a scaled-up version to facilitate an automated method for registering protein images. Until this scaled-up version is operational and applied to a large number of proteins within a family, a definitive conclusion related to correlations between protein function and/or evolution to flexibility characteristics cannot be made. In particular, the biological significance of finding high similarity with respect to different alignments cannot be drawn. Nevertheless, this work demonstrates that methods using image and signal processing provide a sensitive characterization of similarity with regard to flexibility measures that often show only subtle differences. With further analysis, we plan to employ wavelet techniques on additional flexibility measures in order to extract additional information from the images and signals.

## Figures and Tables

**Table:** (a)

Aligned IDF signal distances			
TRX			
	1EP7	1FB6	1THX

1EP7	∗	0.005253	0.007149
1FB6	0.005253	∗	0.009897
1THX	0.007149	0.009897	∗

CheY			
	1TMY	2CHE	3CHY

1TMY	∗	0.003924	0.008473
2CHE	0.003924	∗	0.011775
3CHY	0.008473	0.011775	∗

CaM			
	1EXR	1NIW	1RFJ

1EXR	∗	0.022749	0.028437
1NIW	0.022749	∗	0.024646
1RFJ	0.028437	0.024646	∗

**Table:** (b)

Aligned hydrophobicity signal distances			
TRX			
	1EP7	1FB6	1THX

1EP7	∗	20731.11	18421.28
1FB6	20731.11	∗	17714.09
1THX	18421.28	17714.09	∗

CheY			
	1TMY	2CHE	3CHY

1TMY	∗	17146.56	0.222448
2CHE	0.108108	∗	0.108108
3CHY	0.222448	0.108108	∗

CaM			
	1EXR	1NIW	1RFJ

1EXR	∗	3846.10	4308.31
1NIW	3846.10	∗	3848.70
1RFJ	4308.31	3848.70	∗

**Table 2:** Image-based distances for TRX family.

	1EP7-1FB6	1EP7-1THX	1FB6-1THX
Euclidean distance	0.644481	0.449019	0.458533

Gap measure	0.28787	0.312717	0.394472

**Table 3:** Image-based distances for CheY family.

	1TMY-2CHE	1TMY-3CHY	2CHE-3CHY
Euclidean distance	0.4751	0.318182	0.084265

Gap measure	0.22985	0.22985	0.108108

**Table 4:** Image-based distances for CaM family.

	1EXR-1NIW	1EXR-1RFJ	1NIW-1RFJ
Euclidean distance	0.22816	0.090917	0.332156

Gap measure	0.212416	0.208319	0.292903
